# The impact of nonalcoholic fatty liver disease on chronic viral hepatitis – progression to cirrhosis

**DOI:** 10.1186/1471-2334-13-S1-O27

**Published:** 2013-12-16

**Authors:** Cristina Olariu, Adriana Nurciu, Oana Șchiopu, Maria Nițescu, Dan Olteanu, Mihai Olariu

**Affiliations:** 1 National Institute for Infectious Diseases “Prof. Dr. Matei Balş”, Bucharest, Romania; 2 University Emergency Hospital, Bucharest, Romania

## Background

Nonalcoholic fatty liver disease (NAFDL) is the hepatic manifestation of the metabolic syndrome (MS) and it is associated with obesity, diabetes mellitus and dyslipidemia. We evaluated the impact of NAFLD on chronic viral hepatitis (HBV and HCV) and the progression to cirrhosis for these patients.

## Methods

Between April 2011 and March 2013, 210 consecutive patients with chronic viral hepatitis were submitted. Patients with excessive alcohol consumption (above 20 g/day for males, 10 g/day for females) have been excluded. The steatosis, necroinflammatory activity and fibrosis were estimated using FibroMax (non-invasive liver tests). The serological and virological tests established the diagnosis of chronic viral hepatitis. We divided the patients into 2 groups: patients without steatosis (n=80) and with steatosis (n=130).

## Results

Statistical analysis pointed out the prevalence of female (62.7%); average age 42.3±10.72 years. The presence of steatosis (≥S1 – SteatoTest) was significantly correlated with: female gender (p=0.028), older age (p=0.003) and the degree of hepatic fibrosis (≥F3 – FibroTest, p=0.001).

## Conclusion

The prevalence of steatosis in chronic viral hepatitis was 61.9%. We observed a positive correlation of steatosis with age, female sex and the fibrosis stage. Steatosis was an independent risk factor for progression of the liver disease.

